# Supplementary material for: Impact of timing from last dose of dexamethasone administration to delivery, different steroid courses, and fetal number on preterm neonatal outcomes
Source: PLoS One. 2026 Mar 2;21(3):e0343138. doi: 10.1371/journal.pone.0343138 (PMC12952613; doi:10.1371/journal.pone.0343138)
Supplement: Attachment — Submitted filename: Point by point response to editor and reviewers.docx [file pone.0343138.s001.docx]

**POINT- BY-POINT RESPONSE TO EDITOR AND REVIEWER**

**RESPONSE TO EDITOR**

Comment 1.

When submitting your revision, we need you to address these additional requirements.

1. Please ensure that your manuscript meets PLOS ONE's style requirements, including those for file naming. The PLOS ONE style templates can be found at

<https://journals.plos.org/plosone/s/file?id=wjVg/PLOSOne_formatting_sample_main_body.pdf> and

[https://journals.plos.org/plosone/s/file?id=ba62/PLOSOne_formatting_sample_title_authors_affiliations.pdf](https://track.editorialmanager.com/CL0/https:%2F%2Fjournals.plos.org%2Fplosone%2Fs%2Ffile%3Fid=ba62%2FPLOSOne_formatting_sample_title_authors_affiliations.pdf/1/010f019a4e21d380-928abfba-f006-4314-ab20-bc9311252d34-000000/eosawqGj4XxNknWnnip5o6HUe1YUm_u_gVoKrwwmLDs=235)

Response to editor

Thank you for your guidance. We have revised the manuscript to ensure full compliance with *PLOS ONE* style requirements. The manuscript has been reformatted according to the provided templates, and all file names have been updated to follow the journal’s naming conventions.

Comment 2.

2. Thank you for stating the following financial disclosure:
The Faculty of Medicine Siriraj Hospital, Mahidol University, provided funding support ([IO] R016733004).

Please state what role the funders took in the study. If the funders had no role, please state: "The funders had no role in study design, data collection and analysis, decision to publish, or preparation of the manuscript."
If this statement is not correct you must amend it as needed.
Please include this amended Role of Funder statement in your cover letter; we will change the online submission form on your behalf.

Response to editor

Thank you for your note. The funders had no role in study design, data collection and analysis, decision to publish, or preparation of the manuscript. We have included this statement in the cover letter as requested.

Comment 3.

3. Thank you for stating the following in the Acknowledgments Section of your manuscript:
We thank the Faculty of Medicine Siriraj Hospital, Mahidol University, for financially supporting the editing of this paper by Mr David Park. We also appreciate the administrative support provided by Ms Rangsima Srichai.

We note that you have provided funding information that is not currently declared in your Funding Statement. However, funding information should not appear in the Acknowledgments section or other areas of your manuscript. We will only publish funding information present in the Funding Statement section of the online submission form.
Please remove any funding-related text from the manuscript and let us know how you would like to update your Funding Statement. Currently, your Funding Statement reads as follows:
The Faculty of Medicine Siriraj Hospital, Mahidol University, provided funding support ([IO] R016733004).

Please include your amended statements within your cover letter; we will change the online submission form on your behalf.

Response to editor

Thank you for the clarification. We have removed the funding-related text from the Acknowledgments section. The updated Acknowledgment now reads:

“We thank the Faculty of Medicine Siriraj Hospital, Mahidol University, for the English editing assistance provided by Mr. David Park, and for the administrative support from Ms. Rangsima Srichai.”

The Funding Statement remains as follows:

“The Faculty of Medicine Siriraj Hospital, Mahidol University, provided funding support ([IO] R016733004).”

The updated statements have been included in the cover letter as requested.

Comment 4.

4. Your ethics statement should only appear in the Methods section of your manuscript. If your ethics statement is written in any section besides the Methods, please delete it from any other section.

Response to editor

Thank you for your guidance. The ethics statement has been retained only in the *Methods* section and removed from all other parts of the manuscript, in accordance with *PLOS ONE* requirements.

Comment 5.

5. We note that you have indicated that there are restrictions to data sharing for this study. For studies involving human research participant data or other sensitive data, we encourage authors to share de-identified or anonymized data. However, when data cannot be publicly shared for ethical reasons, we allow authors to make their data sets available upon request. For information on unacceptable data access restrictions, please see <http://journals.plos.org/plosone/s/data-availability#loc-unacceptable-data-access-restrictions>.

Before we proceed with your manuscript, please address the following prompts:

a) If there are ethical or legal restrictions on sharing a de-identified data set, please explain them in detail (e.g., data contain potentially identifying or sensitive patient information, data are owned by a third-party organization, etc.) and who has imposed them (e.g., a Research Ethics Committee or Institutional Review Board, etc.). Please also provide contact information for a data access committee, ethics committee, or other institutional body to which data requests may be sent.

b) If there are no restrictions, please upload the minimal anonymized data set necessary to replicate your study findings to a stable, public repository and provide us with the relevant URLs, DOIs, or accession numbers. Please see <http://www.bmj.com/content/340/bmj.c181.long> for guidelines on how to de-identify and prepare clinical data for publication. For a list of recommended repositories, please see <https://journals.plos.org/plosone/s/recommended-repositories>. You also have the option of uploading the data as Supporting Information files, but we would recommend depositing data directly to a data repository if possible.

Please update your Data Availability statement in the submission form accordingly.

Response to editor

Thank you for your note. There are no ethical or legal restrictions on sharing the de-identified data. The data supporting the findings of this study are available from the corresponding author upon reasonable request. The following statement has been included in the manuscript:

**Availability of data and materials:**
The data supporting the findings of this study are available from the corresponding author upon reasonable request. Researchers may contact the corresponding author directly for data access and further information regarding data sharing arrangements.

Comment 6.

6. If the reviewer comments include a recommendation to cite specific previously published works, please review and evaluate these publications to determine whether they are relevant and should be cited. There is no requirement to cite these works unless the editor has indicated otherwise.

Response to editor

Thank you for the clarification. We have carefully reviewed the publications suggested by the reviewers and have cited only those that are relevant to our study. Irrelevant recommendations were not included, in accordance with *PLOS ONE* guidelines.

**RESPONSE TO REVIEWER 1**

Comment 1.

Line 69-70: though the incidence is not significantly different, there is a more recent data on this published in 2023 (Ohuma, Eric O et al. National, regional, and global estimates of preterm birth in 2020, with trends from 2010: a systematic analysis. The Lancet, Volume 402, Issue 10409, 1261 – 1271)

Response to reviewer

We thank the reviewer for this helpful suggestion. The sentence has been revised to include updated global estimates of preterm birth rates and numbers, as follows:

“In 2014 and 2020, the global preterm birth rate was 10.6% and 9.9%, respectively, accounting for approximately 14.84 million and 13.4 million live preterm births (1, 2).”

We have also added the new reference **Ohuma et al., 2023 (The Lancet)** to provide the most recent global data.

New Corrected data

## Introduction part: Line 68-71. In 2014 and 2020, the global preterm birth rate was 10.6% and 9.9%, respectively, accounting for approximately 14.84 million and 13.4 million live preterm births. (1, 2)

Comment 2

Line 74-75: author should provide a more recent article for reference.

Response to reviewer

We appreciate the reviewer’s suggestion to include a more recent reference. The cited article has been updated accordingly. A recent and relevant reference has been added as follows:

*Costa, F., Titolo, A., Ferrocino, M., Biagi, E., Dell'Orto, V., Perrone, S., & Esposito, S. (2024). Lung Ultrasound in Neonatal Respiratory Distress Syndrome: A Narrative Review of the Last 10 Years. Diagnostics, 14.* [*https://doi.org/10.3390/diagnostics14242793*](https://doi.org/10.3390/diagnostics14242793)*.*

In addition to adding a recent reference, we have also included the original citation that first defined the terminology of respiratory distress syndrome for historical and conceptual accuracy:

*Avery ME, Mead J. Surface properties in relation to atelectasis and hyaline membrane disease. AMA J Dis Child. 1959;97(5, Part 1):517–23.*

New corrected data

Reference 5. *Costa, F., Titolo, A., Ferrocino, M., Biagi, E., Dell'Orto, V., Perrone, S., & Esposito, S. (2024). Lung Ultrasound in Neonatal Respiratory Distress Syndrome: A Narrative Review of the Last 10 Years. Diagnostics, 14.* [*https://doi.org/10.3390/diagnostics14242793*](https://doi.org/10.3390/diagnostics14242793)*.*

Comment 3.

Line 116-121: author should provide better clearance on the definition and/or severity of the neonatal outcomes that were evaluated, or provide references for such. For example, what grade of IVH did they look at? What stage of NEC and grade of BPD did they look at? Define or cite reference for definition of BPD? What maternal complications were evaluated? And so on.

Response to reviewer

In the revised manuscript, we have added detailed definitions and corresponding references for all neonatal and maternal outcomes evaluated.

New corrected data

Part of Material and methods, Line 123-158

- Respiratory distress syndrome (RDS) was diagnosed in preterm infants who presented with clinical signs of respiratory distress, including tachypnea (respiratory rate > 60 breaths per minute), nasal flaring, expiratory grunting, and chest wall retractions and required respiratory support such as continuous positive airway pressure (CPAP), intubation, or surfactant therapy. The diagnosis was confirmed by characteristic chest X-ray findings, including a reticulogranular (ground-glass) appearance and air bronchograms, consistent with surfactant deficiency. (12, 13)
- Transient tachypnea of the newborn (TTNB) is characterized by tachypnea (respiratory rate >60 breaths per minute) developing shortly after birth, typically within the first 6 hours, accompanied by mild respiratory distress (grunting, nasal flaring, or retractions) and radiographic findings consistent with pulmonary fluid retention (e.g., prominent vascular markings, interlobar fissure fluid, or hyperinflation). The condition resolves within 72 hours without evidence of infection or structural lung disease. (14)
- Apnea of prematurity is defined as a cessation of breathing lasting 20 seconds or longer, or a shorter pause accompanied by bradycardia (heart rate <100 beats/min) and/or oxygen desaturation (SpO₂ <80%), occurring in infants born before 37 weeks of gestation after exclusion of other causes such as infection or airway obstruction. (15)
- Intraventricular hemorrhage (IVH) was classified according to Papile’s grading system (grades I–IV) based on cranial ultrasound findings, with clinically significant IVH defined as grade II or higher. (16, 17)
- Necrotizing enterocolitis is diagnosed and staged according to Bell’s classification, with *clinically significant disease defined as stage II or higher*. (18, 19)
- Early-onset neonatal sepsis is defined by sepsis occurring within the first 72 hours of life, confirmed by a positive blood culture or clinical findings consistent with systemic infection (e.g., temperature instability, respiratory distress, or hemodynamic instability). (18, 20)
- Neonatal pneumonia is diagnosed by clinical symptoms (tachypnea, grunting, retractions) plus radiographic evidence of pulmonary infiltrates or consolidation, with or without positive cultures. (21)
- Maternal postpartum complications: (22)

Common complications evaluated include postpartum hemorrhage (blood loss ≥500 mL after vaginal delivery or ≥1,000 mL after cesarean section), postpartum infection (endometritis, wound infection), hypertensive complications, and readmission due to postpartum morbidity.

Comment 4.

Line 125-127: why is chest Xray not included in making diagnosis of RDS

Response to reviewer

The definition of respiratory distress syndrome (RDS) has been revised to include chest X-ray findings as part of the diagnostic criteria. The updated definition now states that RDS was diagnosed based on clinical signs of respiratory distress, requirement for respiratory support or surfactant therapy, and characteristic radiographic findings (reticulogranular pattern and air bronchograms) consistent with surfactant deficiency, in accordance with the European Consensus Guidelines on RDS (Sweet et al., 2019) and Wang et al., 2004.

New corrected data

Part of Material and methods, Line 123-158

Respiratory distress syndrome (RDS) was diagnosed in preterm infants who presented with clinical signs of respiratory distress, including tachypnea (respiratory rate > 60 breaths per minute), nasal flaring, expiratory grunting, and chest wall retractions and required respiratory support such as continuous positive airway pressure (CPAP), intubation, or surfactant therapy. The diagnosis was confirmed by characteristic chest X-ray findings, including a reticulogranular (ground-glass) appearance and air bronchograms, consistent with surfactant deficiency.

Comment 5.

Line 134: author should define the groups before this point

Response to reviewer

We thank the reviewer for the helpful suggestion. The groups mentioned in the sample size calculation have now been clearly defined as **incomplete, complete, and multiple courses of antenatal corticosteroids (ACS)**. The paragraph has been revised accordingly to clarify the basis of group comparison in the sample size estimation.

New corrected data

Part of statistical analysis Page 9, Line 162-172

## Sample size calculation

Our pilot study indicated that 68% of preterm infants with RDS had received a full course of dexamethasone, whereas 50% had received either an incomplete course or multiple courses. To evaluate the impact of these three groups of ACS exposure (incomplete, complete, and multiple courses) on RDS and other outcomes, we calculated the required sample size. We set a significance level of 0.01 (two-sided) and a power of 95%. Using the nQuery Advisor program, we determined that 263 infants with RDS were needed per group. Assuming a 15% incidence of RDS among preterm infants, we required a total of 1753 preterm infants (263 × 100/15). This number was rounded to 1800 to ensure sufficient power to detect differences related to the course of ACS and to accommodate potential variations in the study population.

Comment 6.

Line 147: I would recommend that the authors discuss association of ACS timing with mortality in their cohort

Response to reviewer

We have now analyzed and reported the association between antenatal corticosteroid (ACS) timing and neonatal mortality.

The Results section includes the following statement: “There was no significant difference in neonatal mortality among the groups categorized by the time from the last dexamethasone dose to delivery (P = 0.727).”

The Discussion section has also been updated to note that ACS timing was not significantly associated with neonatal mortality in our cohort, although prior meta-analyses have shown improved survival when delivery occurs within 1–7 days after ACS administration (McGoldrick et al., 2020).

New corrected data

The result part, Line 216-218.

There was no significant difference in neonatal mortality among the groups categorized by the time from the last dexamethasone dose to delivery (P = 0.727) (Table 3).

The discussion part, Line 382-385.

In our cohort, ACS timing was not significantly associated with neonatal mortality, consistent with previous studies showing that while antenatal corticosteroids reduce overall neonatal death, the timing effect within the recommended window may vary depending on gestational age and clinical circumstances. (38)

Comment 7.

Line 158-159 (Table 1): author to define what a complete course of ACS is, or cite a reference for it in the method. How many courses of multiple ACS dose were given? At what number of multiple doses did they start to see harm or no benefit?

Response to reviewer

We have now added a clear definition of the antenatal corticosteroid (ACS) courses in the Methods section, placed before the *Sample size calculation* subsection. A complete course was defined as four 6-mg intramuscular doses of dexamethasone administered every 12 hours (total 24 mg), in accordance with World Health Organization and American College of Obstetricians and Gynecologists (ACOG) guidelines. An incomplete course referred to fewer than four doses, and a multiple course was defined as two complete courses of ACS administered at least 7 days apart in separate episodes of threatened preterm labor.

In our cohort, 168 women (9.3%) received two courses of ACS. No significant increase in adverse neonatal or maternal outcomes was observed among those who received two courses compared with a single complete course. The relevant reference (*Roberts & Dalziel, Cochrane Database Syst Rev. 2006;3:CD004454*) has also been cited.

New corrected data

Methods an material section Line 164-172.

**Definition of antenatal corticosteroid courses**

- A complete course of antenatal corticosteroids (ACS) was defined as four 6-mg intramuscular doses of dexamethasone administered every 12 hours (total 24 mg), according to World Health Organization and American College of Obstetricians and Gynecologists (ACOG) guidelines. (23)
- An incomplete course referred to fewer than four doses administered before delivery.
- A multiple course was defined as two complete courses of ACS given at least 7 days apart in separate episodes of threatened preterm labor.

Comment 8.

Line 237-240: It is expected that infants born very or extremely preterm will need NICU admission as well as most likely need resp support. I do not understand why the author analyzed and reported this.

Response to reviewer

We agree that NICU admission and respiratory support are expected among very and extremely preterm infants. These outcomes were analyzed to provide a **comprehensive overview of neonatal morbidity across gestational age categories** and to enable comparison of outcome trends within our cohort. Reporting these findings also supports **internal consistency** of the dataset by demonstrating expected associations with increasing prematurity.

To clarify this intention, we have revised the Results section to include the following phrase:

“..., as expected due to increasing immaturity, and are presented here for completeness and comparison across gestational age groups.”

New corrected data

Results part, Line 286-287

….. Moderate, very, and extremely preterm births were also significantly associated with TTNB, persistent tachypnea of the newborn, ventilator support, phototherapy, and neonatal intensive care unit admission (*P* < 0.001; Table 6), as expected due to increasing immaturity, and are presented here for completeness and comparison across gestational age groups.

Comment 9.

Line 339-346: this is a report of result. It should therefore be under result, and not discussion.

Response to reviewer

We agree that the text could have been misinterpreted as presenting our own results. We would like to clarify that this paragraph refers to the findings of the **Effective Perinatal Intensive Care in Europe (EPICE)** study, which were cited to compare and contextualize our own results rather than to report data from our study. Therefore, we have **retained this paragraph in the Discussion section** but have **revised the wording** to make clear that it describes **previously published findings** and supports interpretation of our results in the context of existing literature.

New corrected data

…Consistent with our results, the **Effective Perinatal Intensive Care in Europe (EPICE)** study (27) reported that administration of antenatal corticosteroids (ACS) even shortly before delivery was associated with a marked reduction in in-hospital mortality and severe neonatal morbidity among very preterm infants (24–31 weeks’ gestation). The investigators observed that mortality declined by more than 50% between 18 and 36 hours after ACS administration, suggesting that even brief exposure before birth may provide significant survival and health benefits for very preterm infant.

Comment 10.

Line 366-389: I disagree with the authors about these clinical implications conclusion as this is a retrospective cohort study. I think an RCT or a meta-analysis will be needed to make such recommendations.

Response to reviewer

We agree that, given the retrospective cohort design, strong clinical practice recommendations are not appropriate. To address this, the statements regarding clinical implications have been revised to reflect that our findings are observational and should be interpreted with caution. We now emphasize that definitive recommendations would require evidence from randomized controlled trials or meta-analyses. The revised text now highlights potential implications only and acknowledges the need for further prospective validation.

New corrected data

Clinical implication part, Line 423-429.

In singleton pregnancies, our findings suggest that delivery 2–7 days after completing a full course of ACS may be associated with more favorable respiratory outcomes. However, these observations are derived from a retrospective design and therefore may be subject to residual confounding. Prospective studies and randomized controlled trials are needed to confirm whether optimizing timing within this interval leads to improved clinical outcomes.

Comment 10.

PS: It is important for the authors to describe the practice of surfactant administration in their institution, and what effect does it have on the reported results.

Response to reviewer

We have now added a description of our institution’s **surfactant administration protocol** to the **Methods** section to clarify clinical practice. In our setting, **surfactant is administered selectively** to preterm infants diagnosed with **respiratory distress syndrome (RDS)** who require **intubation and mechanical ventilation**, in accordance with the **European Consensus Guidelines on the Management of RDS (Sweet et al., 2019)**.

All infants were managed following this consistent institutional protocol during the study period. Because the **indication and timing of surfactant use were standardized**, we do not expect this practice to have introduced bias or significantly affected between-group comparisons. This clarification has been incorporated into the revised manuscript.

New corrected data

Method section, line 163-173

***Surfactant administration protocol*** *At our institution, surfactant is administered selectively to preterm infants with clinically and radiographically confirmed respiratory distress syndrome (RDS) who require intubation and mechanical ventilation, in accordance with the European Consensus Guidelines on the Management of RDS. (12) Infants who can be managed with continuous positive airway pressure (CPAP) alone do not routinely receive surfactant. All infants included in this study were managed under this same standardized institutional protocol. Because the indication and timing of surfactant administration were consistent throughout the study period, this practice is unlikely to have introduced bias or significantly affected comparisons between groups.*

**RESPONSE TO REVIEWER 2**

Comment 1.

The paper is well written in standard English. The statistical analysis is appropriate, however the primary outcome did not reach statistical significance. The subject population consisted primarily of infants of gestational ages for which ACS are not typically given and many were not admitted to the NICU. The conclusions do not add very much to the field as a result.

Response to reviewer

We acknowledge that the primary outcome (RDS) did not reach statistical significance. However, our study identified significant differences in other clinically relevant respiratory outcomes such as the need for continuous positive airway pressure (CPAP) support and neonatal intensive care unit (NICU) admission, which provide additional insight into the respiratory effects of antenatal corticosteroid (ACS) timing and course.

Regarding the gestational age distribution, we recognize that many infants in our cohort were in the late preterm range, which reflects real-world clinical practice in our tertiary referral center, where ACS administration is extended to pregnancies up to 36 + 6 weeks. This inclusion allows evaluation of ACS effects across a broader gestational spectrum, complementing data from studies that focus mainly on very preterm infants.

To address the reviewer’s concern, we have revised the Discussion to highlight these points, emphasizing that while our findings may not change current practice, they add contextual evidence from a large, mixed-gestational-age population and help identify areas for future randomized or prospective investigation.

New corrected data

Discussion part Line 428-436

***Considerations on Primary Outcome and Study Scope***

Although the primary outcome, respiratory distress syndrome (RDS), did not reach statistical significance among the study groups, several secondary respiratory outcomes, including the need for continuous positive airway pressure (CPAP) support and neonatal intensive care unit (NICU) admission, showed significant differences according to the timing and course of antenatal corticosteroid (ACS) administration. These findings suggest that even in the absence of a clear reduction in RDS incidence, variations in ACS exposure may still influence the severity of neonatal respiratory morbidity.
